# RNA Sequencing Characterizes Transcriptomes Differences in Cold Response Between Northern and Southern *Alternanthera philoxeroides* and Highlight Adaptations Associated With Northward Expansion

**DOI:** 10.3389/fpls.2019.00024

**Published:** 2019-01-28

**Authors:** Dasheng Liu, David Horvath, Peng Li, Wenmin Liu

**Affiliations:** ^1^Shandong Institute of Environmental Science, Jinan, China; ^2^USDA-ARS, Sunflower and Plant Biology Research Unit, Fargo, ND, United States; ^3^College of Life Sciences, Shandong Normal University, Jinan, China

**Keywords:** invasive plant, *Alternanthera philoxeroides*, local adaptation, cold hardiness, RNA sequencing, range expansion, North China

## Abstract

*Alternanthera philoxeroides* recently expanded its range northwards in China. It is unknown if the range expansion has a genetic and/or epigenetic basis, or merely an environmental basis due to a warming climate. To test these possibilities, we used an RNAseq approach with a common greenhouse design to examine gene expression in individuals from the northern edge and central portion of alligator weed range from China to determine if there were differences in their responses to cold temperatures. We hypothesized that if the recent range expansion was primarily environmental, we would observe few differences or only differences unrelated to low-temperature adaptations. We assembled over 75,000 genes of which over 65,000 had long open reading frames with similarity to sequences from arabidopsis. Differences in expression between northern and southern populations that were both exposed to low temperatures showed similar expression among genes in the C-REPEAT/DRE BINDING FACTOR (CBF) regulon. However, gene set and sub-network enrichment analysis indicated differences in the response of photosynthetic processes and oxidative stress responses were different between the two populations and we relate these differences to cold adaptation. The transcriptome differences in response to cold between the individuals from the two populations is consistent with adaptations potentiating or resulting from selection after expansion into colder environments and may indicate that genetic changes have accompanied the recent northward expansion of *A. philoxeroides* in China. However, we cannot rule out the possibility of epigenetic changes may have a role in this expansion.

## Introduction

Biological invasions are becoming a major global environmental and economic problem (Cohen and Carlton, 1998), and intentional or unintentional human activities have increasingly resulted in the introduction of invasive species far outside their native or naturalized range. The fact that invasive species are introduced to new areas that differ from their native range provide a valuable insights into how ecological and evolutionary processes are influenced under novel environmental conditions (Sax et al., 2007; Lachmuth et al., 2010). Understanding the mechanisms driving successful adaptation and invasion in novel environments is a key issue in ecology, evolution, and conservation biology (Vandepitte et al., 2014). Physiological and phenotypic plasticity, evolution, and admixtures have all been linked to local adaptation resulting in range expansion and invasiveness (Bock et al., 2014). Next-generation sequencing is an ideal tool for examining adaptive responses and genetic differences resulting in range expansion and is uniquely suited for non-model species (Prentis et al., 2010; Stapley et al., 2010; Ekblom and Galindo, 2011; Puzey and Vallejo-Marin, 2014). However, sequence evidence of adaptation during the invasion process is currently scant for most invasive plants due to the lack of genomic resources in weedy species (Stewart et al., 2009; Vandepitte et al., 2014).

Recently, the invasive plant, *Alternanthera philoxeroides* (alligator weed), has recently expanded it range northward by nearly 5° of latitude beyond what was its predicted range in China (Liu et al., 2012). This expansion occurred despite evidence that *A. philoxeroides* in China has limited genetic variation(Xu et al., 2003). Thus, this observation offers an opportunity to look for genetic and/or epigenetic factors associated with this recent northward range expansion.

*Alternanthera philoxeroides* weed (*A. philoxeroides*) belongs to family Amaranthaceae (Mabberley, 1997), order Caryophyllales, subclass Caryophyllidae (Cronquist, 1988), and it is an invasive semi-aquatic weed (Julien and Bourne, 1988). It originated in the Parana River region of South America (Maddox, 1968; Vogt et al., 1979), and was spread to the other areas of South America, North America, Asia, Australia and some adjacent island countries (Julien et al., 1995). It is a very difficult to manage invasive weed, and in all cases *A. philoxeroides* has become invasive in its introduced habitats (Julien et al., 1995). This weed grows in both aquatic and terrestrial habitats and can also invade farm lands. Its stems are hollow, buoyant, and its floating mats can expand over surfaces of all types of waterways making them difficult to navigate, clogging drainage canals and waterways, reducing water flow (Buckingham, 2002), and disrupting economies (Holm et al., 1997). *A. philoxeroides* is a polyploid species with a genome size that varies on the ploidy level between 2 and 6 n (Telesnicki et al., 2011). Yet, despite its world-wide invasiveness, less than 100 genes have been sequenced and made public^1^.

*Alternanthera philoxeroides* was introduced to China mainland in 1940 by the Japanese in Shanghai (N31.4, E121.5) (Diao, 1990), and cultivated widely as a forage in southern China in the 1950–60s. It subsequently escaped cultivation and currently inflicts serious damage to agriculture (Yin, 1992; Liu and Huang, 2002), causing annual losses of 600 million Chinese Yuan Renminbi (equivalent to 98 million US dollars) (Li and Xie, 2002). This weed is on the first shortlist of the invasive species requiring special control in China (State Environmental Protection Administration of China and Chinese Academy of Sciences, 2003). A recent survey indicated that *A. philoxeroides* has now invaded the Xiaqing River in Jinan (N36.6, E117.1), 500 km north of the Shanghai in northern China (Liu et al., 2006, 2012). This invasion is more extensive than the 32° north latitude northern range limit predicted for *A. philoxeroides* in China (Julien et al., 1995). It is unknown if the recent ability of this weed to survive winters in this northern region is due to selection for genes that have increased the cold hardiness of this species, or if the range expansion was due primarily to a warming climate.

Increased cold hardiness can result from either avoidance mechanisms such as production of underground propagules or repression of ice nucleation and crystal growth, or from resistance mechanisms such as changes in expression of genes involved in altering membrane lipid saturation, resistance to oxidative stress by reducing reactive oxygen species (ROS) production through reduced photosynthesis from unadapted thylakoid membranes, or induction of free radical scavenging enzymes such as APX1, SOD1, NPX1, etc. (Gusta and Wisniewski, 2013). Many cold tolerant plant species can cold acclimate in response to chilling temperatures (Gusta and Wisniewski, 2013). Induction of these protective responses are orchestrated through several cold-responsive ‘regulons’ (Fowler and Thomashow, 2002). Specific transcription factors such as the C-REPEAT/DRE BINDING FACTOR (CBF) family of transcription factors and the transcription factor RESPONSIVE TO HIGH LIGHT 41 (ZAT12) are often induced upon exposure to chilling temperatures (Thomashow, 2010). These in turn alter the expression of downstream genes required for modifying the cellular physiology to withstand cold. Such co-regulated clusters of genes and their regulators are often referred to as regulons. The best characterized is the CBF2 regulon which can work with or without integrating information from hormones such as abscisic acid (ABA). Another regulon associated with and augmented by subset of cold responses is controlled by the ABSCISIC ACID RESPONSIVE ELEMENTS-BINDING FACTOR (ABRE) family of transcription factors (Shinozaki and Yamaguchi-Shinozaki, 2007). Both the CBF and ABRE regulons are responsive to, and provides feedback to transcription factors that control circadian responses in plants (Dong et al., 2011; Cao et al., 2005). Likewise, ZAT12 regulates a smaller but still important set of genes (including *CBF2*) (Vogel et al., 2005). *ZAT12* is also regulated by circadian genes and in particular blue light signals through CYR1 and CRY2 (Kilian et al., 2007). ZAT12 has been specifically linked to responses involved in high light stress responses (Davletova et al., 2005).

Many of the studies described above utilized microarray analysis for gene expression resulting from cold treatments. However, the reduced cost of next generation sequencing has made it possible to combine transcriptomics analysis with gene sequencing and discovery in non-model systems such as wild invasive weeds. Indeed, recent studies on cold stress in tea (*Camellia sinensis*) (Wang et al., 2013), and *Chrysanthemum nankingense* (Ren et al., 2014) highlight the power of this new technology for identifying cold-responsive genes. Because such studies produce large numbers of gene sequences, they also provide a source of gene sequences from non-model plants suitable for phylogenetic analyses (Puzey and Vallejo-Marin, 2014). Additionally, such sequence databases provide a rich source of genetic markers including potential simple sequence repeats (SSRs) and single nucleotide polymorphisms (SNPs).

Here we examine the differences in cold-responsive gene expression in *A. philoxeroides* between three individuals each from two populations, one from the northern edge of its range in Jinan, North China and the other from the central portion of its range in Shanghai, South China. If the recent range expansion was primarily environmental, we would expect few differences in gene expression between cold-treated plants from the northern and central populations, or only differences unrelated to low-temperature adaptations. We test this hypothesis by looking for differences in transcriptome responses indicative with a response to cold using a common garden experimental design. Thus, any such differences, if observed, would be attributed to genetic and/or epigenetic differences between the individuals from the two populations. Additionally, we expected to develop a database of expressed sequences tags (ESTs) for *A. philoxeroides* and eventually identify polymorphisms that have potential as genetic markers for more cold-adapted populations.

## Materials and Methods

### Plant Material

50–60 individual wild *A. philoxeroides* plants were collected in November 2013 from local rivers of Jinan and Shanghai. Jinan, the provincial capital of Shandong, is located in North China in the lower reaches of the Yellow River, the second longest river in China. The average annual temperature in Jinan is 14.7°C with an average of -0.4°C in January and 27.5°C in July. The average annual rainfall is 672.7 mm. Its location is N36.6, E117.1. Shanghai is located in South China in the lower reaches of the Yangtze River, the longest river in China. The average annual temperature in Shanghai is 16.6°C with an average of 4.7°C in January and 28.0°C in July. The average annual rainfall is 1184.4 mm. Its location is N31.4, E121.5. The mentioned meteorological data from China Meteorological Data Sharing Service System (cdc. cma.gov. cn), and also see our paper (Liu et al., 2012).

The plants were collected from each location and established in a common garden plot of greenhouse in Jinan, Shandong province, North China. Plants were grown hydroponically in the 67 × 43 × 7 cm pots containing 50% concentration Hoagland’s solution and watered every 2–3 days with same solution (Hoagland, 1938). Plants were allowed to grow in the greenhouse with night and day temperatures of 16–31°C, respectively, under natural light condition and photoperiod for 4 weeks prior to treatment. Plants were first subjected to 2 day of interim temperatures (11–19°C night/day) in a non-temperature controlled greenhouse with natural light condition and photoperiod in the Jan 2014, and then moved to an unheated open-air concrete building and subjected to 2 weeks of cold temperatures (daytime 6–13 C, nighttime 4–9 C) with natural lighting supplemented during the day with incandescent lighting to increase ambient light intensity within the building. Three representative individual *A. philoxeroides* plants were selected from each location, and the top 2nd and 3rd leaf pairs were excised between 10:00 am–12:00 am and immediately frozen in liquid nitrogen for future RNA extraction and sequencing library construction.

### RNAseq Library Construction and Sequencing

A total amount of 3 μg RNA per sample was used as input material for the RNA sample preparations. Sequencing libraries were generated using NEBNextUltra RNA Library Prep Kit for Illumina (NEB, United States) following manufacturer’s recommendations and index codes were added to attribute sequences to each sample. Briefly, mRNA was purified from total RNA using poly-T oligo-attached magnetic beads. Fragmentation was carried out using divalent cations under elevated temperature in NEBNext First Strand Synthesis Reaction Buffer (5X). First strand cDNA was synthesized using random hexamer primer and M-MuLV Reverse Transcriptase RNaseH-. Second strand cDNA synthesis was subsequently performed using DNA polymerase I and RNase H. Remaining overhangs were converted into blunt ends via exonuclease/polymerase activities. After adenylation of 3’ ends of DNA fragments, NEBNext Adaptor with hairpin loop structure were ligated to prepare for hybridization. In order to select cDNA fragments of preferentially 150∼200 bp in length, the library fragments were purified with AMPure XP system (Beckman Coulter, Beverly, United States). Then 3 μl USER Enzyme (NEB, United States) was used with size-selected, adaptor-ligated cDNA at 37°C for 15 min followed by 5 min at 95°C before PCR. Then PCR was performed with Phusion High-Fidelity DNA polymerase, Universal PCR primers and Index (X) Primer. At last, PCR products were purified (AMPure XP system) and library quality was assessed on an Agilent Bioanalyzer 2100 system.

The clustering of the index-coded samples was performed on a cBot Cluster Generation System using TruSeq SR Cluster Kit v3-cBot-HS (Illumina) according to the manufacturer’s instructions. After cluster generation, the library preparations were sequenced on an Illumina Hiseq 2000/2500 platform and 100 bp paired-end reads were generated.

### Sequencing Analysis and Bioinformatics

Primer sequences were trimmed from raw sequence reads and the resulting files were trimmed for high quality reads using the program Sickle-quality-based-trimming (Joshi and Fass, 2011) in the iPlant discovery environment (DE) (Oliver et al., 2013). The trimming parameters were: quality scores greater than 20 with a minimum trimmed read length of 70 bases. The trimmed reads from each end for all six samples (from both populations) were concatenated and the two files (one from each paired end) were kmer normalized using the Trinity_normalize_by_kmer_coverage (Grabherr et al., 2011) in the iPlant DE with parameters of 30 reads per kmer. The resulting two files were assembled using the program Trinity (Robertson et al., 2011) in the iPlant DE using the default parameters. The assembled contigs were checked for quality by assessing contig length and coverage and for completeness using the program CEGMA (Parra et al., 2007) in the iPlant DE. Likewise, the reads from each of the three individuals from the two populations were similarly assembled and assessed separately. BlastX against the arabidopsis peptide database was used to annotate the assembled contigs with a minimum blast hit of*E*-value < 10^-5^.

The program Bowtie (Langmead et al., 2009) was used to map the reads from the individual samples back to the Trinity assembled reference contig file. Likewise, the reads were also mapped separately to the Trinity assembled reference files for both the central and northern populations accordingly. Differential expression analysis was accomplished using the program RSEM (Li and Dewey, 2011) with the program EBseq to identify differentially expressed genes (Leng et al., 2012). Programs were run on the iPlant Atmosphere resource using an Ubuntu 12.04.5 - iPlant Base interface and scripts with options are noted in Supplementary Table S1. The differentially expressed genes and contigs were additionally annotated by BlastX against the non-redundant database (downloaded May 23, 2013) using the Blast 2.26 stand-alone program (McGinnis and Madden, 2004). Open reading frames were identified using the program Transcript decoder 1.0 in the iPlant DE. Gene set and sub-network analysis of the resulting normalized expression vales of the genes was done using the program Pathway Studio 9.0 (Bogner et al., 2011). Only expression data from contigs with >10 transcripts per million (TPM) in all three biological replicates from at least one of the treatment groups, and which had significant similarity (as above) to arabidopsis genes were used for gene set enrichment analysis or sub-network enrichment analysis (GSEA or SNEA). Raw sequence data, normalized expression data, and transcript assemblies are all available through the Gene Expression Omnibus (accession # GSE63585) and links therein.

## Results

### Sequencing and Assembly of Transcripts

Our study presents the first transcription sequence for *A. philoxeroides*. Over 212 million one hundred base, paired end RNA sequencing reads were obtained from 3 cold-treated individuals from both central and northern populations. Sequences ranging from 29 and 48 million fragments were obtained after trimming the raw reads based on sequence quality scores with a minimum read length of 70 bases. Fewer than 8% of the fragments were removed by the quality trimming (Table 1). Assembly of the combined sequences by the program Trinity resulted in over 700,000 contigs (transcript equivalents) representing over 360,000 components (gene equivalents) with an N50 of over 1300 bases and a maximum length of over 16,000 bases (Table 2). A comparison to a list of highly conserved eukaryotic genes using the program Core Eukaryotic Genes Mapping Approach (CEGMA) indicated that over 93% were represented as full length transcripts in the assembly with over 99% represented at least by partial sequences (Table 3).

**Table 1:** Numbers or raw and trimmed reads from the six different libraries.

Library	Raw reads	Trimmed reads
Northern 1	37394696	34672020
Northern 2	31662866	29386538
Northern 3	33400004	31161772
Central 1	36996538	34551382
Central 2	37294423	34744435
Central 3	36135432	33623100
Total	212883959	198139247


**Table 2:** Assembly statistics from the three assemblies.

Count	sum_len	N50	min_len	max_len	med_len	ave_len	sd_len	Data source
745348	634400797	1387	201	16184	507	851	850	Combined populations
574621	485190870	1395	201	16710	495	844	845	Northern population only
582659	488823500	1382	201	16946	493	839	843	Central population only


*Count = number of contigs, sum_len = total number of bases assembled, N50 = the size in bases above which half of the transcriptome is represented in contigs equal to or larger than the indicated value, min_len = size of smallest contig length, max len = size of largest contig, med_len = median contig length, ave_len = average contig length, sd_len = the standard deviation of contig lengths, data source = the data set used to assemble the contigs.*

**Table 3:** Results of completeness study using the CEGMA program.

Assembly	% complete	% partial
Combined	93.95	100
Northern only	96.37	100
Central only	97.58	99.6


*Statistics of the completeness of the genome based on the percentage of 248 Conserved Eukaryotic Genes that are represented among assembled transcripts as either full length (complete) or as fragments of transcripts (partial).*

Similarly, assemblies were performed using the transcripts from each of the two populations separately. Both separate assemblies resulted in over 570,000–580,000 contigs representing about 290,000 components each, with N50s and maximum contig lengths very similar to the combined assembly (Table 2). Likewise, results from the program CEGMA indicated similar completeness of coverage of the conserved genes obtained for both separate assemblies (Table 3).

### Annotation of the Assemblies

**FIGURE 1:**
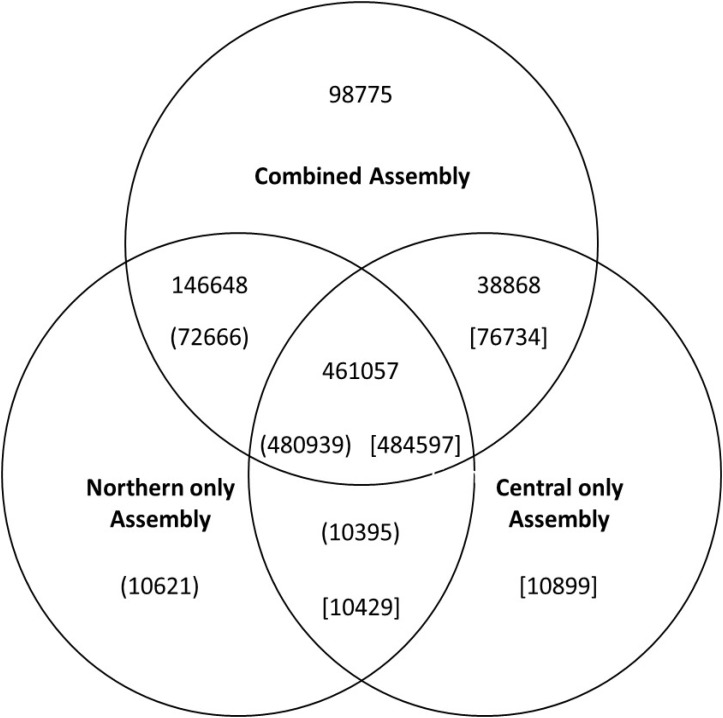
Venn diagram showing the numbers of uniquely assembled contigs in each of the three assemblies and the number of contigs which were assembled in more than one assembly process. The numbers in parenthesis or brackets indicate the number of commonly assembled contigs identified from reverse BlastN analysis. The differences in numbers for each comparison is because some individual contigs had best matches multiple contigs in the various analyses.

Contigs were examined to identify those containing long open reading frames indicative of functional genes. We identified 330,642 transcripts with reading frames over 90 amino acids in length in the combined 745,348 contigs assembly. These 330,642 transcripts represented about 75,000 genes. Of these, 58,000 were also assembled from both population-specific sequences as determined by aligning the resulting assembled contigs using BlastN of. Overlap between the different the contig assembles can be visualized in the Venn diagram (Figure 1). Of the 75,000 open reading frame-containing transcripts, 64,000 had BlastX hits (E < 10^-5^) to the arabidopsis TAIR10 database. These blast hits were used for the primary functional annotation of the transcripts (Supplementary Table S1). BlastX against the non-redundant database was performed for differentially expressed transcripts and genes (Supplementary Tables S2, S3) as well as from a random set of 4000 open reading frame-containing contigs. Of the 5,429 differentially-expressed transcripts for which a source organism was identified by BlastX against the non-redundant database, 4241(77%) had the top hit corresponding to a known plant gene, 267 had a top hit corresponding to an animal gene (247 of which were to arthropods), 11 were likely bacterial genes, 6 were from plasmodium 3 from protists, and 1 from fungi. If the gene list was trimmed to include only those transcripts that had expression values greater than 10 transcripts per million (TPM) in all three biological replicates of either of the two treatments (populations), over 99% of the differentially accumulating transcripts with species information were annotated as plant genes (Supplementary Tables S2, S3). Far fewer non-plant hits with only 2% of non-plant origin (data not shown) were observed in a random set of 1000 open-reading-frame-containing transcripts with hits to the non-redundant database than from the differentially expressed set.

### Differential Gene Expression

Differential gene expression was examined using the program RSEM to quantify and normalize the expression of both transcripts and genes using each of the assembled transcript files as reference transcriptomes. 6,200 differentially expressed transcripts and 851 differentially expressed genes were identified when the combined assembly was used as the reference (Supplementary Tables S2, S3). Of the significantly differentially expressed sequences, 46% of the contigs and 33% of the genes were more highly expressed in the northern populations in response to cold temperatures. We also examined the differential expression using separate assemblies from either the northern or central populations (Supplementary Table S1). When fragments were mapped to the assembly constructed only from sequences derived from the central population, we observed 4,566 differentially expressed genes with only 66 genes also identified as being differentially expressed when the transcripts were mapped to the combined assembly. Likewise, 7,318 genes were differentially expressed when transcripts were mapped to the northern population assembly of which 1,653 were also significantly differentially expressed in the combined assembly. Of the 66 genes common to the mapping to both the central population assembly and the combined assembly, only 18 were found to be differentially expressed when mapped to all three assemblies. Among the differentially expressed sequences, approximately 251 transcripts and 12 genes encoded putative transcription factors.

### Gene Set Enrichment Analysis

**FIGURE 2:**
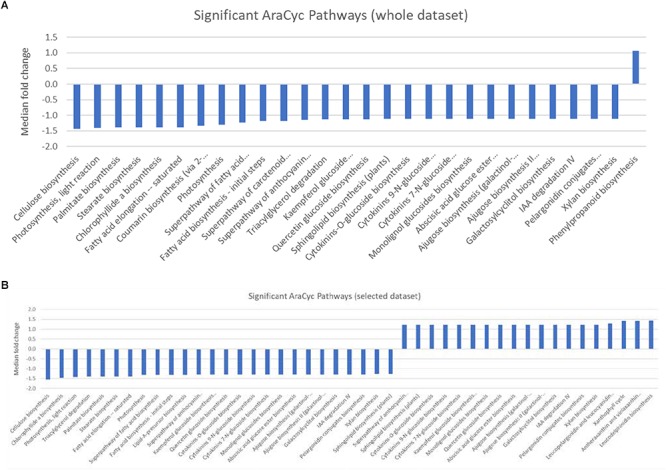
**(A,B)** Graphic showing the median fold change of genes associated with significantly over-represented AraCyc pathways identified from **(A)** either the entire dataset, or **(B)** selected genes that were preferentially expressed in either the central population relative to the northern population (positive values) or genes that were preferentially expressed in the northern population (negative values).

**FIGURE 3:**
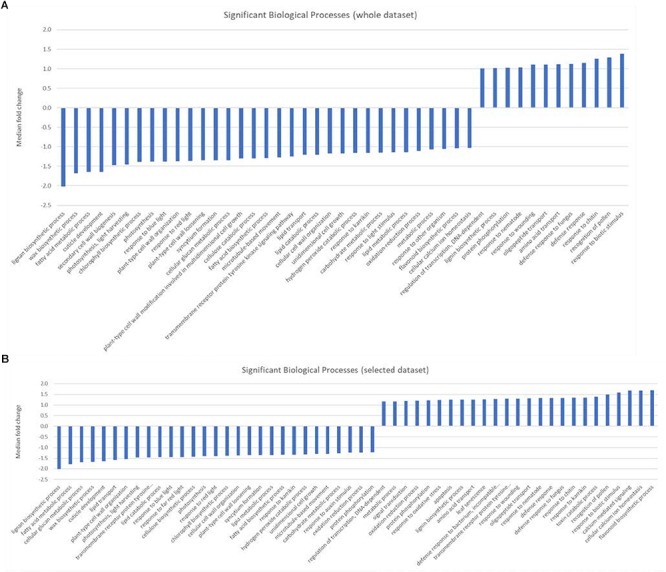
**(A,B)** Graphic showing the median fold change of genes associated with significantly over-represented biological processes identified from **(A)** either the entire dataset, or **(B)** selected genes that were preferentially expressed in either the central population relative to the northern population (positive values) or genes that were preferentially expressed in the northern population (negative values).

Gene set enrichment analysis (GESA) was used to identify over-represented terms from the AraCyc (Müller et al., 2003) and GO (Ashburner et al., 2000) ontologies for those genes with arabidopsis homologues and with expression values greater than 10 TPM in all three biological replicates from either treatment group. A full list of all the enriched terms with *p* values < 0.05 can be found in Supplementary Table S4. We identified 28 significantly over-represented terms (*p* < 0.001) associated with biochemical pathways from the AraCyc database when the entire gene set was subjected to GSEA (Figure 2A). All but one of these (phenylpropanoid biosynthesis) had median expression values that suggested they were more highly expressed in the central population. Likewise 19 and 26 over-represented AraCyc terms were identified when genes were filtered for those that were preferentially expressed in the northern or central populations, respectively (Figure 2B). An analysis of GO terms associated with biological processes identified 45 significantly over-represented terms for which 33 had median expression values of the associated genes indicating preferential expression in the central population (Figure 3A). Again, 25 and 29 biological process terms were over-represented among genes that were preferentially expressed in only the northern or central populations, respectively (Figure 3B).

### Sub-Network Analysis

**FIGURE 4:**
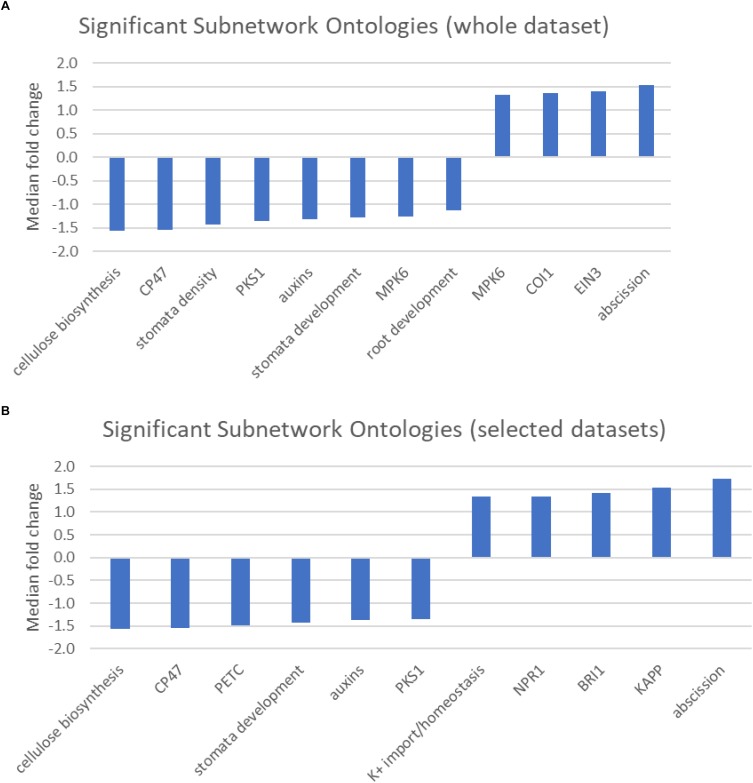
**(A,B)** Graphic showing the median fold change of genes associated with significantly over-represented Sub-network ontologies identified from **(A)** either the entire dataset, or **(B)** selected genes that were preferentially expressed in either the central population relative to the northern population (positive values) or genes that were preferentially expressed in the northern population (negative values).

The pathway studio program maintains a database of transcription factor targets, interacting proteins, and proteins/chemicals regulating cellular processes (Supplementary Table S5). The results of the sub-network analysis identified a limited number of regulatory or protein interaction networks associated with differences in response to cold stress between the two populations (Figure 4A,B). Even with a stringent significance cut off of *p* < 0.01, we identified 12, 6, and 5 terms when all of the genes were analyzed or when the genes were limited to those preferentially expressed in the central and northern populations, respectively.

## Discussion

### Sequencing and Assembly Characterize Numerous Genes From Invasive *A. philoxeroides*

We performed an RNAseq analysis following a cold treatment on three individuals of two *A. philoxeroides* populations. One population from Jinan in northern China and appear to have gained the ability to flourish in a more northern climates previously uninhabitable by this weed. The other individuals were from a central population near Shanghai in the center of its established range. The goals of these experiments were to develop a sequence database of genes from *A. philoxeroides*, and to determine if these two populations differ in their transcriptomic response to cold temperature treatments. The cold treatments did not appear to result in obvious physical differences between the two populations, but transcriptome differences were detected between the tested individuals from the two populations. Further studies are needed to determine if either population is better able to survive and reproduce in the more northerly latitudes. However, differences in gene expression were observed between the individuals from these two populations and the changes in gene expression are consistent with cold resistance mechanisms. These observations are most consistent with the hypothesis that the northward expansion of *A. philoxeroides* into Northern China is not simply the result of global warming, but is likely due to evolution of adaptations to colder environments.

We identified over 75,000 genes with long open reading frames of which >85% were significantly similar to known arabidopsis genes. BlastX searches against the non-redundant database of the differentially expressed transcripts (5,429) indicated that ∼25% might result from contamination of the samples from other organisms (mostly arthropods). A search of the literature suggests that most RNAseq assemblies contain about 1–3% non-plant sequences, although one study in rubber tree (*Heveabrasiliensis*) indicated that 17% of the assembled transcripts were from non-plant sources (Mantello et al., 2014). However, the percentage of non-plant genes identified from differentially expressed contigs, as was done in our case, is likely to be over-represented. Low abundance genes such as those likely coming from contaminating organisms such as insects, bacteria and fungi, are more likely to be classified as differentially expressed (Trapnell et al., 2013). This possibility is supported by an assessment of non-plant transcripts from a random sample of 1000 open-reading-frame-containing transcripts with hits to the non-redundant database from the combined assembly in which only 2% were suspected as coming from non-plant sources. Regardless, our results provide a rich set of gene sequences from this invasive weed which could be used as source for SSR or SNP markers for further population genetic analyses.

Although a majority of the genes from the combined assembly were represented in assemblies generated from the individual populations, there were clearly unique contigs that were assembled from a single population. Some of these are likely to be poorly represented sequences, perhaps from contaminating DNA from non-*Alternanthera philoxeroides* sources or from otherwise low abundant sequences. This hypothesis is bolstered by the fact that, of the 1978 contigs from the complete assembly that were represented only in the assembly of the central population, only 128 had expression levels >10 TMP in all biological replicates of either treatment group (Supplementary Table S1). Surprisingly, 258 of these 1978 transcripts were classified as differentially expressed with 4 transcripts expressed at >10 TPM in all three biological replicates from the northern population. The later observation suggests that even though these genes were not assembled in the northern population assembly, a modest number of RNAseq reads from the northern samples still mapped to the assembled contigs. This observation underscores the potential problem of false positives resulting from differential mapping rather than true differential expression when comparing gene expression among two different accessions.

### Differential Expression Suggests Genetic Changes Provide Enhanced Cold Tolerance of Individuals From Northern Populations

Because of the observed high number of differentially expressed non-plant transcripts we limited further GSEA and SNEA to a subset of genes with >10 TPM and which has reliable BlastX hits to known arabidopsis genes. A large number of genes were identified as differentially expressed (*q*-value < 0.05) between the central and northern populations in response to the cold treatment. The most notable differences were involved in photosynthesis, which appear repressed in the tested individuals from northern populations. This observation is supported from both an examination of the most differentially expressed genes and from the gene set and sub-network enrichment analyses. One of the primary causes of damage due to chilling stress is the production of oxidative radicals produced when the electron transport chain is disrupted by altered membrane fluidity that prevent proper association of the proteins involved in the electron transport in the chloroplast stroma (Wise, 1995). One mechanism through which plants survive cold treatments is by modifying or repressing the photosynthetic apparatus under chilling conditions. In another study, two isogenic lines of wheat in which a single point mutation resulted in reduced cold tolerance (Wells et al., 1969) were used in a transcriptomics analysis analogous to our study on *A. philoxeroides* (Sutton et al., 2009). A comparison of the GSEA results from differences between the two lines following cold treatment also implicated altered photosynthesis as potentially underlying the differences in cold tolerance (Karki et al., 2013).

In our study, very little evidence points to alterations in the CBF regulon between the northern and central populations. Neither “neighbors of CBF” or “response to cold” were indicated as over-represented ontologies by SNEA or GSEA. Likewise, no known targets of CBF were indicated as significantly differentially expressed – despite the presence of putative sequences of targets (such as a *COR47*-like gene) among the contigs and genes that were assembled. However, we observed up-regulation of other cold-responsive regulons in the northern population. Genes such as a *COR27*-like and *RAV1*-like that in arabidopsis are modulated through circadian clock and ABA signaling (deMontaigu et al., 2010) were found to be significantly up-regulated in northern populations. This observation is consistent with alterations in upstream genes involved in responses that integrate ABA and circadian signaling required for non-CBF-regulated cold-induced gene expression in the northern populations.

In addition to non-CBF regulated cold inducible circadian-responsive genes, GSEA identified numerous genes in biotic defense responses, such as salicylic acid and flavonoid biosynthesis, as up-regulated in the northern population. These processes have been associated with growth inhibition in Arabidopsis and other plants (Besseau et al., 2007; Rivas-San Vicente and Plasencia, 2011We have changed “Rivas-San Vicente and Plasencia, 2001” as “Rivas-San Vicente and Plasencia, 2011” inside the text as per the reference list. Kindly confirm if this is fine.). Conversely, processes linked to photosynthesis and growth such as cellulose biosynthesis, photosynthesis - light reaction, and fatty acid biosynthesis were over-represented among genes preferentially expressed in the tested central population individuals. As noted above, photosynthesis during cold treatment is associated with production of damaging oxidative radicals (Wise, 1995). Likewise, continued growth in the presence of oxidative radicals could result in increased damage to DNA (Cadet et al., 2003). Thus, although no obvious CBF regulon modification are indicated, a potentially protective down-regulation in photosynthetic activity and an increase in pathways that are protective to reactive oxygen species are observed in the northern population individuals but not the central population individuals.

## Conclusion

We have demonstrated there are differences in gene expression associated with response to cold treatments between tested individuals from populations of *A. philoxeroides* in the central portion of its range in South China and individuals from a population in North China which has recently expanded its range northward. These results are most consistent with the development and/or selection of genetic and/or epigenetic differences that enhance the cold-tolerance of the northern populations. Further work using a population genetics approach will be needed to determine if the differences observed in these individuals is representative of the whole population, and to identify loci associated with these differences. However, the differences we observed in gene expression point towards alteration in the cold-responsive regulon controlling circadian/ABA signaling rather than alterations in the CBF regulon. Importantly, our results also provide the first large-scale database resource of *A. philoxeroides* gene and transcript sequences. Such sequences should serve as a rich source for markers needed to examine the population genetics of a species that has proven invasive on at least three continents.

## Author Contributions

LD, LP, and LW performed the experiments and collected samples. DH performed primary data analysis and carried out bioinformatics analysis. LD and DH designed the experiments and wrote the manuscript. All authors read and approved the final manuscript.

## Conflict of Interest Statement

The authors declare that the research was conducted in the absence of any commercial or financial relationships that could be construed as a potential conflict of interest.

## Footnotes

1 http://www.ncbi.nlm.nih.gov/gquery/?term=Alternanthera+philoxeroides (accessed December 02, 2014).

## Supplementary Material

TABLE S1 Annotation and expression analysis of all assembled contigs from both the combined assembly and from the assembly of the transcriptome from the three plants collected from the northern and southern populations separately.

TABLE S2 Annotation and gene expression analysis of the assembled contigs of transcripts determined to be differentially expressed between the northern and southern populations.

TABLE S3 Annotation and gene expression analysis of the clustered contigs of genes determined to be differentially expressed between the northern and southern populations.

TABLE S4 Gene set enrichment analysis from the indicated sub-populations of genes.

TABLE S5 Sub-network enrichment analysis from the indicated sub-populations of genes.
